# Trends in Physical Activity Research on Tobacco and/or Alcohol: A Bibliometric Analysis

**DOI:** 10.3390/healthcare13050529

**Published:** 2025-02-28

**Authors:** Antonio Castillo-Paredes, Pablo del Val Martín, Gerson Ferrari

**Affiliations:** 1 Escuela de Ciencias de la Actividad Física, el Deporte y la Salud, Universidad de Santiago de Chile (USACH), Santiago 9170022, Chile; acastillop85@gmail.com; 2 Facultad de Educación y Ciencias Sociales, Observatorio Chileno de Educación Física y Deporte Escolar, Universidad Andres Bello, Las Condes, Santiago 7591538, Chile; pablo.delval@unab.cl

**Keywords:** physical activity, tobacco, smoking, alcohol consumption, behavioral risk factors

## Abstract

**Background/Objectives**: Physical activity allows the enjoyment of personal health benefits in those who practice it, including the possibility of modifying behavioral risk factors such as tobacco and alcohol consumption. These risk factors are responsible for the development of non-communicable diseases, which are preventable and controllable. The scientific field on this object of study has grown in recent years. The main objective of this study was to perform a scientific mapping to explore the trend of annual publications, and to analyze and identify the thematic categories, the authors, countries and journals with the highest number of papers, the most referenced papers and authors, and the most used keywords in research related to physical activity and tobacco and/or alcohol consumption. **Methods:** Through a bibliometric analysis based on traditional bibliometric laws on the scientific documentation related to the subject and indexed in the Main Collection of the Web of Science. The DeSolla Price Law was used to analyze the trend of annual publications, using the coefficient of determination R^2^. Lotka’s law was applied to identify prolific authors, Bradford’s law to highlight the most frequent publication sources, the h-index to identify the most cited articles and Zipf’s law to highlight the keywords most used in research. **Results:** A total of 538 documents were analyzed. The trend followed by annual publications is in an exponential growth phase. Adrian Taylor and Michael Ussher were identified as prolific authors. USA and *Preventive Medicine* were the country and journal with the highest number of publications. The most frequently used words were physical activity, smoking, exercise, alcohol, obesity, and smoking cessation. **Conclusions:** This bibliometric review identified an exponential growth from 1994 to date of research related to physical activity and tobacco and/or alcohol consumption. It allowed us to identify trends and guide the development of future research in these or new related areas.

## 1. Introduction

Tobacco and alcohol are risk factors associated with the development of acute or chronic non-communicable diseases (NCDs) in the world population and, in combination with other risk factors such as poor diet and physical inactivity, are responsible for 41 million deaths annually through cardiovascular diseases, cancers, respiratory diseases, and diabetes. These risk factors, responsible for the development of NCDs, are a major challenge for public health worldwide. Currently, strategies for prevention and control are focused decreasing or delaying their appearance, allowing for the possibility of developing awareness in the population through the promotion of healthy lifestyles, as well as attainable and accessible identification and treatment of these risk factors for the benefit of people, among others that need to be addressed and that are related to economic burden [1]. These prevention actions, getting people to stop smoking and stop drinking alcohol, could contribute to the prevention of cardiovascular and cerebrovascular diseases and the prevention of NCDs associated with tobacco and alcohol consumption, among others [2,3].

The combination of these two risk factors, smoking and drinking alcohol, leads to an increased likelihood of death from all causes [4]. This is because those who are active smokers in combination with the acquisition of unhealthy eating habits develop a higher risk of stroke, an increase in body mass index, and, among other associated diseases, the development of NCDs [5]. Unfortunately, however, it has been shown that those who develop this risk factor are often influenced by those closest to them; for example, if a non-smoker develops a smoking habit, it is frequently because it is observed from a family member or friend [6]. Also, an increase in tobacco consumption generates physical deterioration in people, increasing the risk of physical disability, primarily in men compared to women [7]. On the other hand, excessive alcohol consumption causes damage to the body, including damage to the gastric system, the development of cancers, mental health issues, and the development of physical diseases such as oral cavities and pharynx, esophagus, liver, and colorectal diseases [8,9,10]. Although pharmacological approaches to tobacco and alcohol prevention have been evidenced [11,12], there are also non-pharmacological approaches such as physical activity (PA) as a complementary treatment for tobacco and alcohol control [13,14].

From the point of view of research, academia, and public policy, the definition of physical activity is any generation of movements of different intensities performed by people through their musculoskeletal system, as these movements generate energy demands depending on the activities performed by people in their daily life activities; these include moving from one place to another, walking, and cycling, among others that allow for the fulfillment of personal or social objectives, oriented towards health, labor, or recreational and collective participation [15]. The benefits of PA are numerous; among them, we find a positive association as those who perform PA have a lower risk of all-cause mortality [16,17]. A review of the literature showed that the practice of PA on a regular basis allows for the possibility of preventing different types of cancer, including those associated with overweight and physical inactivity [18]; this review presented the benefits of physical activity in the reduction or prevention of diseases such as cancer incidence, linking sedentary behavior and cancer incidence and obesity and cancer incidence, showing that biological mechanisms linked to physical inactivity, sedentary behavior, and obesity are correlated with cancer risk. Thus, an increase in PA levels contributes to the prevention of NCDs, which are the leading causes of death in the world population due to cardiovascular diseases, metabolic diseases, respiratory diseases, cancers, cognitive diseases, and mental health [19].

On the other hand, there are numerous systematic reviews that have delved into specific aspects of the subject [20,21,22,23,24]. In addition, we found that other researchers used other types of reviews to explore this field of knowledge; this is the case of narrative reviews [25] and scoping reviews [26,27]. In contrast, to the best of our knowledge, there is no bibliometric review in the scientific literature that has analyzed publications related to physical activity and smoking or alcohol consumption. In this way, the development of bibliometric reviews allows the assessment and measurement of the information obtained related to the results of the areas of knowledge to better verify the lines of research in a particular area [28,29,30,31,32,33]. In this way, the development of the bibliometric review allows us to show the scientific productivity of a particular or specific area and therefore the contribution to these areas of knowledge for future decision-making related to trends, research groups, researchers, keywords, and countries with higher productivity in the area, among other relevant information for the strengthening of an emerging or established research area of interest, in order to contribute to the development of the research topic, strengthening it and its social contribution. Considering the above, the development of a bibliometric review of publications related to physical activity and tobacco and/or alcohol consumption would allow us to analyze and explore the development and state of progress of this topic, allowing us to identify the main trends, authors, countries and most influential journals, as well as the possible gaps in knowledge that could guide the development of future research and promotion and prevention strategies that are optimal and effective in the population. Therefore, this study is the first bibliometric analysis of documents published in journals indexed in the Web of Science (WoS). Based on the above, the following research questions have been posed: is the research in a phase of exponential growth, is there a group of authors, countries, and journals that accumulate the largest number of documents, who are these prolific authors and which of them stand out for the impact of their works, and what are the keywords most used by researchers? Therefore, the objectives of the present research were as follows: (1) To analyze and explore the trend followed by the annual publications. (2) To analyze and identify the thematic categories, authors, journals, and countries with the highest number of documents, as well as the most referenced articles and authors. (3) To analyze and identify the keywords most frequently used in research on physical activity and tobacco and/or alcohol consumption.

## 2. Materials and Methods

### 2.1. Database

This study conducted a review of the scientific literature published in journals indexed in the main collection of the Web of Science (WoS), limiting the search to three databases: Expanded (SCIE), Social Science Citation Index (SSCI), and Emerging Sources Citation Index (ESCI); these three collections have been used because they belong to the area of knowledge to be addressed, and in addition, previous works have methodologically stated the use of these collections. For this purpose, a bibliometric analysis was performed based on the traditional laws of bibliometrics [28,29,30].

The WoS main collection was used as the data source. This database is one of the most prestigious databases in the scientific community, given the high standards to which the journals indexed in it are subjected. By choosing this database, we aimed to ensure that all the documents analyzed had passed peer review before being published, in addition to following homogeneous quality criteria and indexes. In addition, WoS is one of the databases most widely used by researchers to perform bibliometric analyses due to its extensive and detailed information included for all indexed documents [31,32,33].

Two researchers (A.C.-P. and P.dV.M) conducted a search in WoS, using the following inclusion criteria: Articles and Reviews of articles whose title contained the terms “Physical Activity” AND (“tobacco” OR “smok*” OR “alcohol consumption” OR “alcohol use”) published in journals indexed in the main collection of WoS. The following exclusion criteria were applied: Meeting Abstracts, Letters, Editorial Managers, Corrections, Notes, and Book Reviews. After searching and downloading the databases in XLS (Microsoft Excel) and plain text without format, both researchers checked that they had reached the same number of documents and unified both databases. In case of discrepancies between researchers, G.F. was in charge of making the decision on the inclusion or exclusion of a document. No time, language, or other limitations were used in the search. This search was conducted on 15 April 2024, including documents indexed up to the time of the search.

### 2.2. Statistical Analysis

A bibliometric analysis is a study method that allows the exploration and analysis of large volumes of scientific data, providing knowledge about the evolution of a field of study, the most important authors and production networks, the most important publication sources involved in the subject, the most cited documents, the countries with the highest number of documents, and the most explored thematic lines, among many other useful insights for the scientific community [34]. This descriptive bibliometric study was based on the traditional laws of bibliometrics and followed the following steps:

(a) The Analyze Reports tool of WoS was used to check the total number of documents, the oldest document, and the number of documents contributed by the search terms.
(b) The Analyze Results tool of the WoS was used to obtain the number of annual publications resulting from the search. The trend followed by these publications was plotted and the coefficient of determination adjusted to an exponential growth ratio (R^2^), which is used as a goodness-of-fit measure and was calculated using Microsoft Excel software [35]. With this procedure, we analyzed whether the trend of the publications was in an exponential growth phase, applying the DeSolla Price-Dobrov Law [36,37,38]. For a better reading and understanding of the results obtained, the calculation of the R^2^ or coefficient of determination makes it possible to evaluate the trend followed by the data analyzed (applying the DeSolla Price-Dobrov law); on this occasion, it is the scientific production related to the study variables. This is a line which allows the identification of growth patterns, making it possible to show whether it is in growth, decline, or recession.
(c) The number of citations received by each of the documents in the main WoS collection was analyzed. To identify the most cited documents, the h documents with number of citations h or greater were highlighted, applying the Hirsch Index (h-index) [31,39,40]. The application of the h-index was plotted in Microsoft Excel, identifying the cut-off point between the number of documents and the number of citations [35].
(d) The Analyze Reports tool of WoS was used to analyze the thematic categories to which the documents were related.
(e) The names of the co-authors were standardized, eliminating duplicities, and a count was made of the relationship of co-authors and the number of documents published by each one of them, using Microsoft Excel to graph this relationship [35]. Lotka’s Law was applied to identify prolific co-authors [41,42,43]. VOSviewer 1.6.20 software [44,45,46,47,48] was used to perform and plot a co-authorship analysis with prolific co-authors: Unit of Analysis (Authors); Normalization (Method: Association Strength; Attraction = 6; Repulsion = 0; Resolution = 1.0). We identified clusters of collaboration and checked the mean year of publication of each author. Prolific co-authors were crossed with co-authors of the most cited papers to identify prominent co-authors, prolific co-authors with one or more papers among the most cited papers [49,50,51].
(f) The countries of the co-authors were analyzed, identifying the countries with the highest number of publications and international collaboration networks. For this purpose, VOSviewer was used [48], performing and plotting a co-authorship analysis [52,53]: Unit of Analysis (Countries); Normalization (Method: Association Strength; Attraction = 8; Repulsion = 0; Resolution = 0.5. Minimum cluster size = 3). We identified collaboration clusters and checked the average year of publication for each country.
(g) Bradford’s law of concentration of science was used to identify the journals that made up the publication core [40,54,55].
(h) Finally, the author keywords were analyzed. Microsoft Excel was used to plot the relationship between the keywords and the number of uses of these in the documents analyzed. Zipf’s law was applied to identify the most used keywords [56,57,58]. Once the most frequently used keywords were identified, they were normalized and a co-occurrence analysis was performed, identifying thematic clusters and checking the mean year of publication of the documents containing each of them, in addition to generating a density map according to the use of these keywords. VOSviewer [48] was used for this purpose: Unit of Analysis (Countries); Normalization (Method: Association Strength; Attraction = 8; Repulsion = 0; Resolution = 1.0).

## 3. Results

### 3.1. Documents Found and Publication Trends

**Figure 1:**
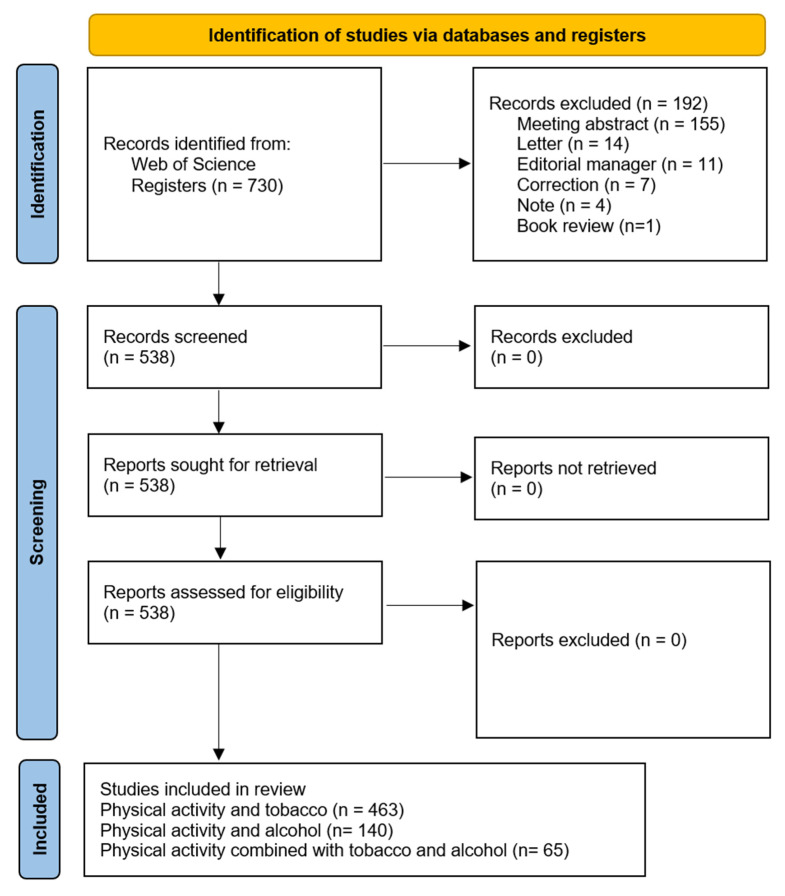
Flow chart of the process of inclusion and exclusion of articles.

Figure 1 shows the flow chart resulting from the search process and application of the inclusion and exclusion criteria, going from 730 initial documents to a total of 538 final documents (504 articles and 34 article reviews). When the searches were conducted separately, a greater number of papers related to physical activity and smoking (463 papers) were found than those related to physical activity and alcohol (140 papers). Merging both searches identified 65 papers that met both conditions.

### 3.2. Publication Trend

**Figure 2:**
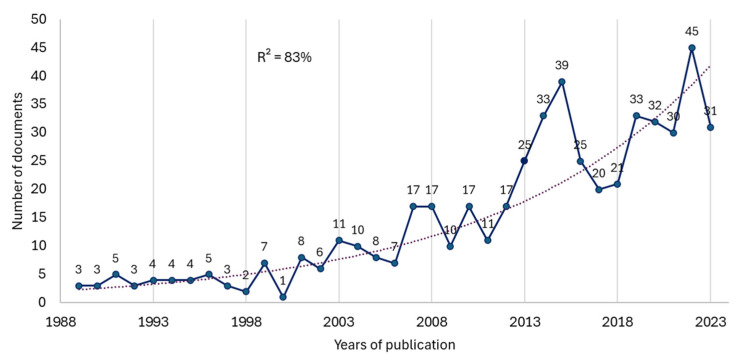
Graph showing the trend of annual publications between 1989 and 2023.

The first papers dated back to 1965 and 1966 [59,60]. However, there was no continuity in annual publications until 1989. From 1989 to the present, at least one paper per year has been published (Figure 2), and the field is currently in a phase of exponential growth (R^2^ = 83%). We searched from 1989 to 2023, excluding 2024 as data for the complete year are not available.

### 3.3. Most Cited Papers

**Figure 3:**
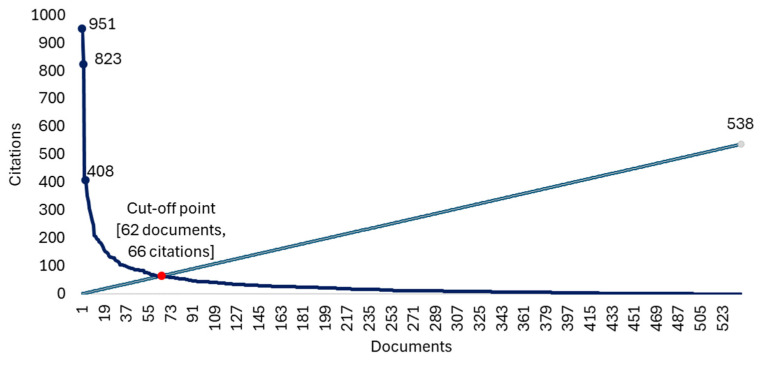
H-Index graph.

Sixty-two papers were found with 66 or more citations, these being the most cited papers (Figure 3). The three most cited papers were: “Longitudinal tracking of adolescent smoking, physical-activity, and food choice behaviors” [61], a 1994 paper with 951 citations, “Depression, Anxiety and Stress during COVID-19: Associations with Changes in Physical Activity, Sleep, Tobacco and Alcohol Use in Australian Adults” [62], a 2020 paper with 823 citations, and “Maintaining mobility in late life. II. smoking, alcohol-consumption, physical-activity, and body-mass index” [63], a 1993 paper with 409 citations. All information concerning the 62 most cited papers is presented in Supplementary Materials S1.

### 3.4. WoS Categories

The documents were related to 63 WoS thematic categories. The thematic category with the highest number of related documents was “Public Environmental Occupational Health” (182 documents). Other prominent subject categories were Medicine General Internal (59 documents), Substance Abuse (48 documents), Psychology Clinical (37 documents), and Psychiatry (31 documents). Other subject categories that made up the top ten categories were Nutrition Dietetics, Oncology, Sport Sciences, Multidisciplinary Sciences, and Environmental Sciences (20–29 documents).

### 3.5. Prolific Co-Authors

**Figure 4:**
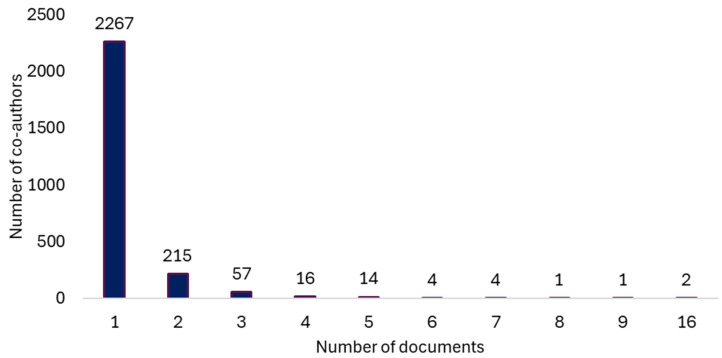
Distribution of authors according to the number of co-authored papers.

We found 2581 co-authors with one or more papers; the range of papers was between 1 and 16 (Figure 4).

**Figure 5:**
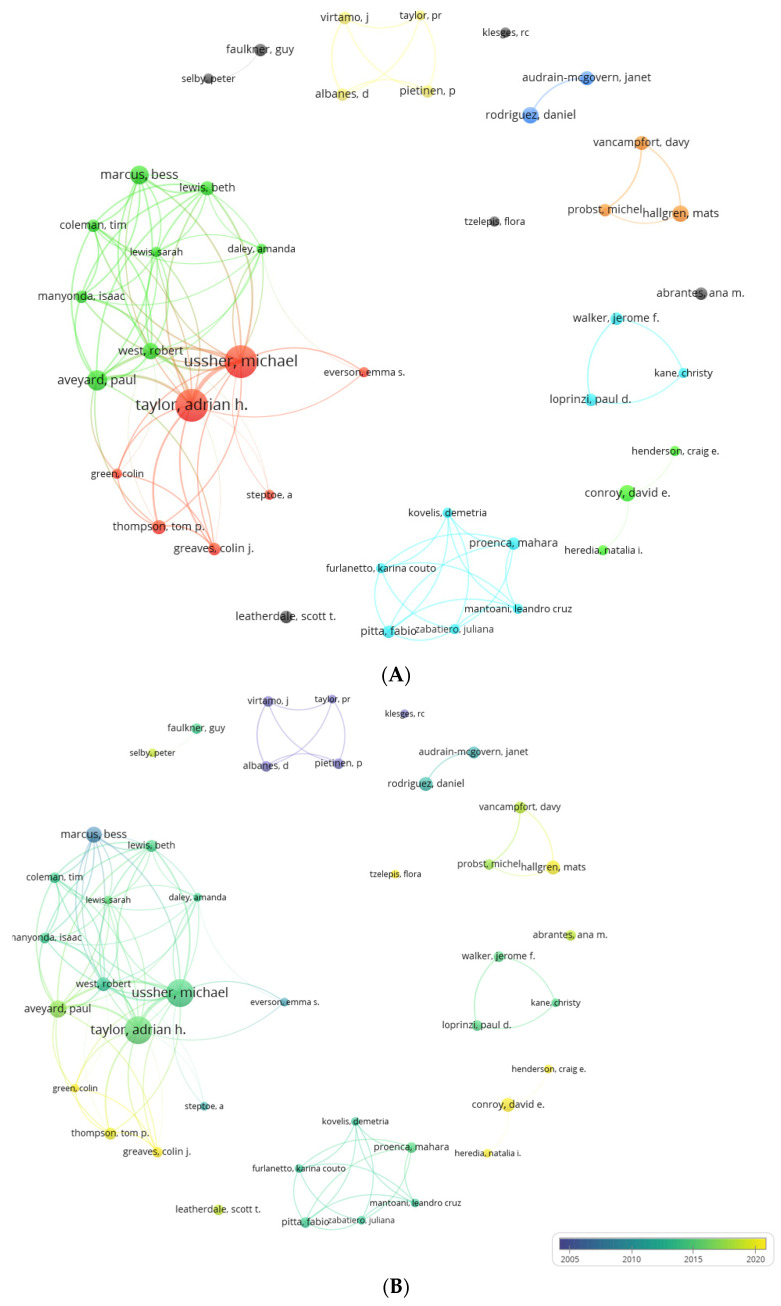
(**A**) Collaborative network of prolific co-authors. (**B**) Collaboration network of prolific co-authors and year of publication of each co-author. Node size is a function of the number of documents. The thickness of the lines is a function of the number of co-authored papers. The color is a function of the average year of publication of each author’s papers.

A total of 42 prolific co-authors were identified, all of them with five or more documents (Figure 5A,B). Prominent among these authors were Adrian Taylor and Michael Ussher (16 documents, 253 and 262 citations, respectively). These co-authors formed a collaborative network with five other prolific co-authors (network cluster). In turn, these two authors were linked to another collaborative network formed by eight other prolific co-authors such as Paul Aveyard (9 documents, 151 citations) and Marcus Bess (8 documents, 449 citations), a prominent co-author with 2 papers among the most cited, among others (green cluster). Another large collaborative network with six prolific co-authors was found, with Fabio Pitta and Mahara Proenca (5 documents, 88 and 82 citations) standing out in this network. Another important collaborative network (yellow cluster) was formed by P. Pietinen (5 documents, 524 citations, 3 documents among the most cited), D. Albanes and J. Virtamo (5 documents, 302 citations, 2 documents among the most cited), and P. Taylor (4 documents, 277 citations, 2 documents among the most cited), all of them considered prominent co-authors. Other prominent, prolific co-authors with one paper among the most cited were D. Rodriguez (7 documents, 167 citations), D. Vancampfort (6 documents, 161 citations), J. Audrain (6 documents, 164 citations), M. Probst (5 documents, 191 citations), R. Klesges (5 documents, 149 citations), and F. Tzelepis (4 documents, 125 citations).

### 3.6. Countries

**Figure 6:**
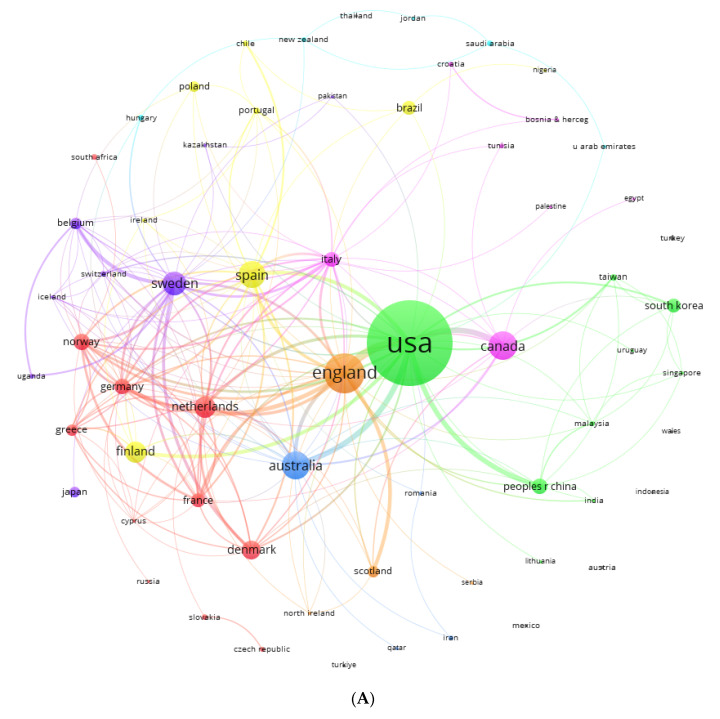

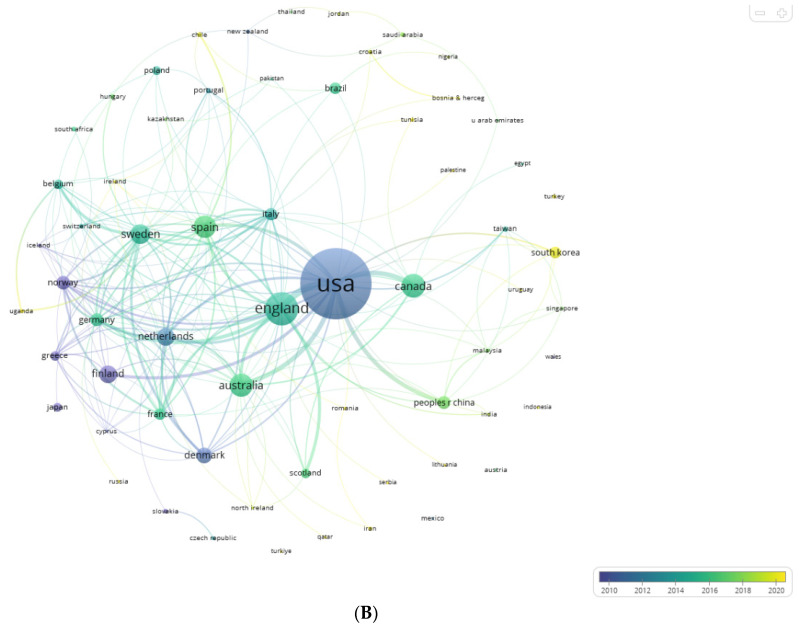
(**A**) International collaboration network by country. (**B**) International collaboration network by country. Score: average publication years.

Eight major international collaboration networks were found with a total of 63 countries (Figure 6A,B). The largest collaborative network (11 countries) was formed by European countries such as the Netherlands (26 documents, 979 citations), Denmark (21 documents, 1117 citations), Norway (17 documents, 1556 citations), and Germany (16 citations, 427 citations), together with seven other countries (red cluster). Another large collaborative network (9 countries) was formed around the country with the highest number of documents and citations (green cluster), the USA (202 documents, 7837 citations). In addition to the USA, this collaborative network was formed with Eastern countries such as China (16 documents, 205 citations), South Korea (14 documents, 100 citations), and Taiwan (5 documents, 449 citations). Other collaborative networks were formed around countries such as Spain (yellow cluster, 8 countries), Sweden (purple cluster, 8 countries), and Canada (pink cluster, 7 countries). England (64 documents, 1935 citations) was the country with the second highest number of documents and citations, forming a small collaborative network together with Scotland, Northern Ireland, and Serbia (orange cluster).

### 3.7. Journals

The papers analyzed were published by 304 journals, with a publication range between 1 and 21 documents. The Bradford core consisted of 16 journals (Table 1). These journals accounted for 5% of the total number of journals, accumulating a total of 153 documents (28%).

Table 2 shows the 16 journals with the highest number of documents. The journals with the highest number of publications were *Preventive Medicine* (21 documents, 1052 citations), *BMC Public Health* (17 documents, 387 citations), and the *International Journal of Environmental Research and Public Health* (17 documents, 939 citations). Within the Bradford core, the journal with the highest ratio of normalized citations per document was the *American Journal of Public Health* (237 citations per document), the journal where the most cited paper was published [61].

### 3.8. Author Keywords

**Figure 7:**
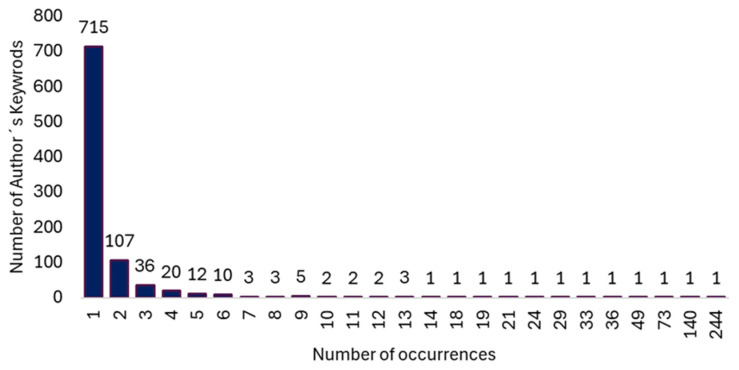
Histogram showing the relationship between the number of keywords as a function of the number of co-occurrences.

We found 932 keywords used by co-authors in the set of documents with a range of occurrence between 1 and 244. By applying Zipf’s law, the 29 most used keywords stood out (Figure 7).

**Figure 8:**
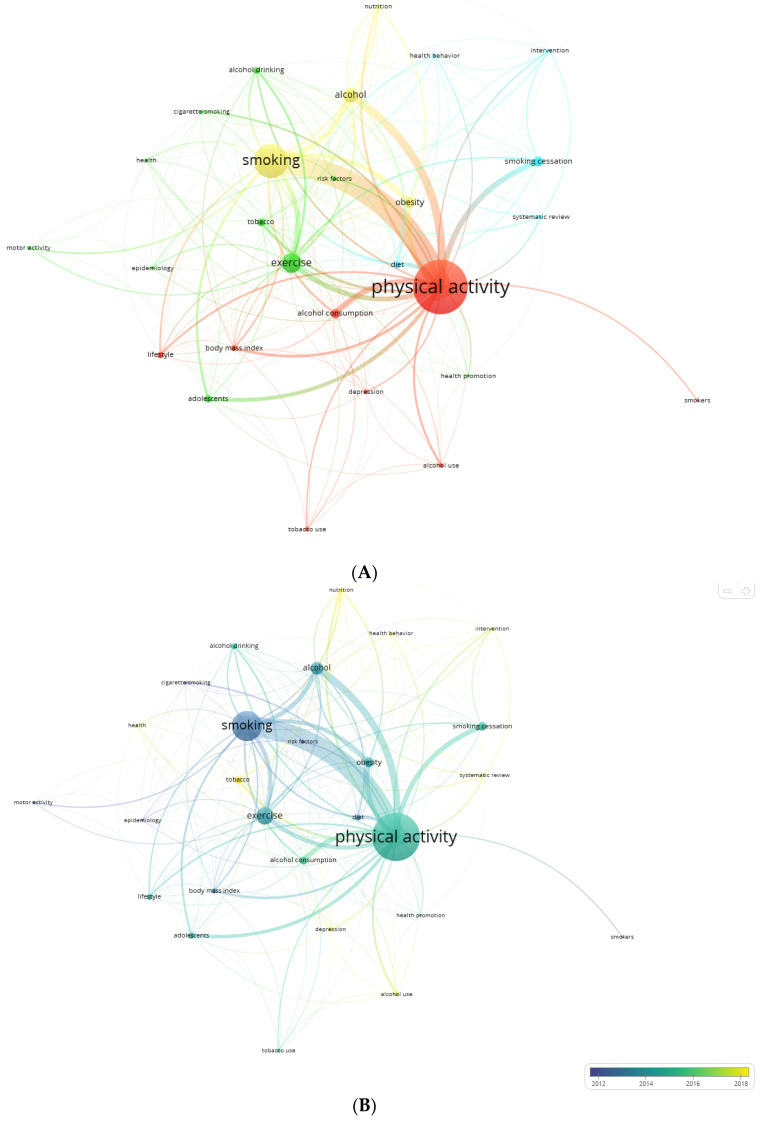

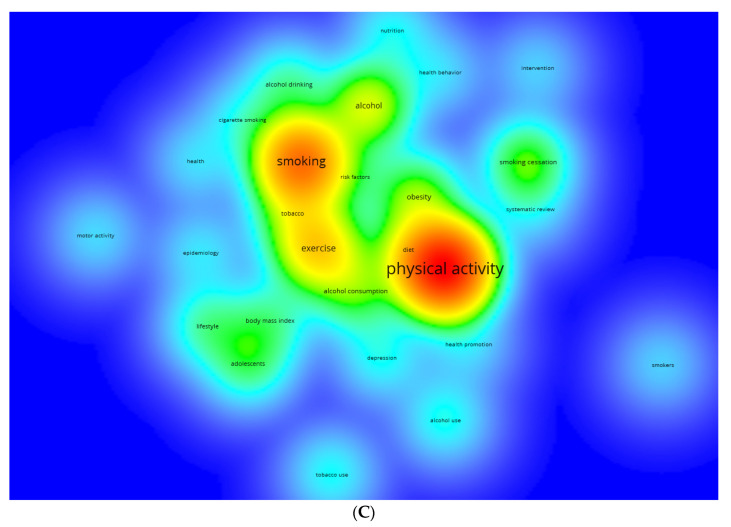
(**A**) Network of most used keywords. Physical Activity (244 occurrences), smoking (140 occurrences), exercise (73 occurrences), alcohol (49 occurrences), obesity (36 occurrences), and smoking cessation (39 occurrences) were the most used keywords. (**B**) Keyword network by average year of publication of documents for each keyword. The size of the node is a function of the number of documents. The thickness of the lines is a function of the number of documents in which they concur. The color is a function of the average year of publication of the documents in which they appear. (**C**) Density map indicates the density of documents for each keyword. The larger the size and the more vivid the color, the more frequent the use.

The keywords “adolescent” and “adolescents” were merged into the term adolescents, as with the keywords “drinking” and “alcohol drinking”, which were merged into the term “alcohol drinking”. Figure 8A–C show the co-occurrence graph with the 27 most used keywords, including the thematic clusters (Figure 8A), the mean year of publication of documents for each keyword (Figure 8B), and the density map (Figure 8C). Four thematic clusters were formed: red (physical activity, tobacco, alcohol, lifestyles and depression), yellow (tobacco, alcohol, obesity and nutrition), green (adolescents, health, exercise, as well as tobacco and alcohol use terms) and blue (smoking cessation, healthy behaviors, diet and intervention). Depression, healthy habits, and nutrition were the keywords with the most recent average year of publication, being more current trends.

## 4. Discussion

This study is the first bibliometric review of research published in journals indexed in the main collection of the Web of Science related to physical activity and tobacco or alcohol. The main objectives of this research were to analyze and explore the trend followed by annual publications, to analyze and identify the thematic categories, authors, journals, and countries with the highest number of publications, as well as the most referenced documents and authors, and to analyze and identify the keywords most used by authors in research on physical activity and tobacco and/or alcohol consumption. In this way, a total of 538 documents were analyzed, checking at what stage of the development of science the object of study was at this time, the thematic categories that grouped the greatest number of documents, the prolific and prominent co-authors, the journals with the greatest number of documents on the object of study, the most cited articles, and the keywords most used by the authors.

### 4.1. Annual Growth in Publications

Our first research objective was to analyze the trend followed by annual publications related to the subject. Although the first two articles on this subject date from 1965 and 1966 [59,60], the publication of papers has been increasing exponentially from 1989 to 2023. Although these findings provide an overview of the progress of this topic in the scientific community, the most recent research with the highest number of citations corresponds to the year 2020 [62,64,65]. In addition, the development of PA interventions is highly recommended worldwide [66,67]. Regarding one of our research questions, are publications on the study target in an exponential growth phase? We found that the publication of scientific articles on this subject has indeed been growing exponentially, taking as a reference the growth since 1989 with 195 citations [68]. This finding ensures that there is a large critical mass of researchers currently developing the object of study. In the last 5 years, a total of 171 articles have been published on the subject, with the year 2022 being the year with the highest number of documents published in journals compared to the corresponding years within this time range. This could be due to the confinement between 2019 and 2020 and the controlled return to normality in many countries from 2021. This is because workplaces and their workers and family groups and their members have had to modify their physical work, leisure, or study spaces and organize them again considering the post-COVID-19 normality and the possible emerging challenges that condition the workplace, the home, and people’s well-being [69]. On the other hand, this increase could be related to the millennium goals, the Sustainable Development Goals (SDGs), and the change towards the 2030 goals because researchers are developing new lines of research considering the social demands, obtaining available resources and global actions towards the promotion of health in the world population [42].

### 4.2. Categories and Prolific Researchers

In relation to our second research objective, which was to identify the thematic categories that accumulated the largest number of documents related to this topic, we were able to identify categories related to “Public Environmental Occupational Health”, “Medicine General Internal”, “Substance Abuse”, “Psychology Clinical”, “Psychiatry”, “Nutrition Dietetics”, “Oncology”, “Sport Sciences”, and “Multidisciplinary Sciences and Environmental Sciences”, with “Public Environmental Occupational Health” being the category which obtained the highest number of documents (182) published on the subject, with the article by Kelder et al. [61] having the highest number of citations (951 citations).

It should be noted that the possibility of identifying the categories of the topics for the development of the research allows us to highlight the inequality that could exist in the field of knowledge, and on the other hand, it also allows us to evaluate the areas of preference for the development of the research [70,71,72].

Regarding our research questions of are there a group of authors, countries, and journals that accumulate the largest number of papers and who are these prolific authors and who among them are prominent, two authors prominent in the research area of the study topic, Adrian Taylor [73] and Michael Ussher [74], were identified. On the other hand, a total of 15 more prolific co-authors collaborating among different research networks were identified, such as Paul Aveyard, Marcus Bess, Fabio Pitta, Mahara Proenca, P. Pietinen, D. Albanes, Virtamo, P. Taylor, D. Rodriguez, D. Vancampfort, J. Audrain, M. Probst, R. Klesges, and F. Tzelepis [75,76,77,78,79,80,81]. In terms of countries and journals, the countries with the most collaborations, published articles, and citations were the Netherlands, Denmark, Norway, and Germany (in Europe), the United States (in the Americas), and China, South Korea, and Taiwan (in Asia), which were the countries with the largest international collaboration networks. On the other hand, as for collaborative countries, these may be the most influential due to the signing of international treaties and economic support from the public and private sector for disease surveillance and treatment and for the advancement of health-oriented scientific production; they are those who have the greatest economic resources for these areas, with the United States being the country with the greatest investment in health and research [82,83]. In addition, the identification of researchers in a particular area allows the recognition of advances in this field of knowledge, evidencing the quality of research, methodological rigor, advances in the area of knowledge, and leadership and validity in the management of projects or research work [84,85].

As for the scientific journals, there were a total of 16 journals, with the journals with the highest number of papers published on the subject being *Preventive Medicine*, *BMC Public Health*, *International Journal of Environmental Research and Public Health* and *Plos One*, compared to the journals that obtained the highest number of citation of the published papers were *American Journal of Public Health* [61], *American Journal of Clinical Nutrition* [86], and *Preventive Medicine* [24]. As for the journals, it has been shown that those that have hybrid access have more possibilities of being cited or consulted compared to those journals that have the subscription modality, even though both pay-per-article models have a discipline related to clinical medicine [87,88,89].

### 4.3. Use of Keywords

Finally, our third research objective, which was to analyze the keywords used by the authors and identify which were the most used and how their use has been trending. The keywords most used in the documents identified on the research topic were physical activity, tobacco, alcohol, lifestyles, depression, and obesity and nutrition, adolescents, health, exercise, tobacco and alcohol consumption, smoking cessation, healthy behaviors, diet and intervention, depression, healthy habits, and nutrition, the latter keywords being the most used in recent times [62,63,90].

However, it is important to remember that the use of the correct words of the study variables for the keywords, the title, and the abstract allow us to accurately identify the content and its positioning for the evaluation of new areas of knowledge that have not been addressed or those that allow us to support the relevance of the content of the research to be developed. In this sense, our study identified the most frequently used terms, although given the great peripheral dispersion of terms with low frequency of use, other emerging terms might not be identified. Thus, some powerful lines of research related to the subject were not reflected in these most frequently used terms. For example, concepts such as COVID-19, sleep or lung cancer were not highlighted, being current research trends.

#### 4.3.1. Limitations and Strengths

This bibliometric study has limitations, and among them we find that only one database was analyzed, which is the most complete and previously used in other studies of this nature. Another limitation is related to the availability of the information in the database, which is limited to the use of the correct words available in the title, abstract, or keywords. In relation to the strengths, we found that this is the first study of this nature, which gives us an overview of research related to physical activity and tobacco or alcohol; in addition, it provides an overview of the number of citations, journals in which these documents were published, most used keywords, and collaboration networks between researchers and countries, which could allow us to pool criteria based on the experience and importance of the research topics. Another strength of this work is that it facilitates decision-making or the development of guidelines with the results obtained because this work provides information related to the researchers in this area of study and their respective institutions and countries, which are relevant for the investment of human or economic resources for collaborative and strategic work in the development of this area of study.

#### 4.3.2. General Practical Applications, Suggestions and Future Lines of Research

The development of this work could allow researchers in this field to establish common criteria for the development and proliferation of research on physical activity and tobacco or alcohol. In this way, a common vision of experts in collaboration with research networks could allow the establishment of a consensus at the moment of the use of key words, specialized journals on the subject, and existing collaboration networks for the development of effective and pertinent research according to the social context. For the development of future research on this methodology developed in this bibliometric re-view, the use of other databases such as PubMed, Scopus, Cochrane, and Embase, among others, is suggested. Another important point is the use of keywords. This bibliometric review allowed us to evidence a high use of keywords, of which a small number were the most used. This could allow a more precise identification of the research if specialized thesauri in this area were used, for example “MeSH” from the National Library of Medicine or the DeCS/MeSH Thesaurus, which are Health Sciences Thesaurus of the Latin American and Caribbean Center for Health Sciences Information of the BIREME and PAHO/WHO. In this way, a consensus would be achieved among researchers for the identification of articles dealing with this topic.

## 5. Conclusions

The research on physical activity, tobacco, and alcohol has grown exponentially since the first articles in 1965 and 1966, with notable progress from 1989 to 2023. Seventeen researchers from sixty-three countries collaborated internationally, and three journals had the highest citations per document. Four journals published fifteen related articles. Key terms included physical activity, smoking, exercise, alcohol, obesity, and smoking cessation. This study highlights emerging research areas, knowledge gaps, and scientific productivity, fostering collaboration between researchers and institutions. It also supports strategic planning and the development of scientific policies to strengthen knowledge and address this critical public health issue.

Finally, in terms of future academic implications, the results of this research allowed the identification of trends in the use of emerging keywords related to the relationship between physical activity, tobacco, and alcohol consumption. This could mean an opportunity for the identified collaborative networks to engage in the exchange and generation of strategic alliances related to the production of scientific evidence located according to the prevailing knowledge of each social and cultural context. In this way, the possibility of joining criteria, identifying possible scientific gaps, could strengthen and consolidate the work of researchers or emerging research nuclei on the subject.

In addition, in terms of empirical implications, the results can be used for the development and design of public policies or non-governmental agreements through strategic, intersectoral, and international planning that allows the creation of strategies located according to the social and cultural context for the promotion of healthy lifestyles and the prevention of these habits that are harmful to the health of the population.

## Figures and Tables

**Table 1:** Bradford’s zones.

Zone	Number of Documents on Thirds (%)		Journals (%)		Bradford Multipliers	Journals (Theoretical Series)	
CORE	153	28%	16	5%		16 × (n0)	16
ZONE I	164	30%	67	22%	4.2	16 × (n1)	60
ZONE II	221	41%	221	73%	3.3	16 × (n2)	224
TOTAL	538	100%	304	100%	3.7		300
						% Error	1.3%

**Table 2:** Physical activity, smoking, and alcohol core journals.

Publication Titles	Doc.	Citations	Norm. Cit.
*Preventive Medicine*	21	1052	50
*BMC Public Health*	17	387	23
*International Journal of Environmental Research and Public Health*	17	939	55
*Plos One*	15	460	31
*American Journal of Clinical Nutrition*	10	1350	135
*Nicotine & Tobacco Research*	10	195	20
*Addictive Behaviors*	9	158	18
*American Journal of Health Promotion*	7	174	25
*BMJ Open*	7	154	22
*Cancer Epidemiology Biomarkers & Prevention*	7	360	51
*American Journal of Epidemiology*	6	869	145
*American Journal of Public Health*	6	1423	237
*Health Psychology*	6	209	35
*Cancer Causes & Control*	5	536	107
*International Journal of Cancer*	5	181	36
*Journal of Adolescent Health*	5	306	61
Doc. (Number of documents); Norm. Cit. (Normalized Citations: Citations/Documents)			

## Supplementary Materials

The following supporting information can be downloaded at: https://www.mdpi.com/article/10.3390/healthcare13050529/s1, Supplementary Material S1: Most cited papers.
