# Supplementary material for: Trends in Physical Activity Research on Tobacco and/or Alcohol: A Bibliometric Analysis
Source: Healthcare (Basel). 2025 Feb 28;13(5):529. doi: 10.3390/healthcare13050529 (PMC11898995; doi:10.3390/healthcare13050529)
Supplement: Supplementary file 1 [file healthcare-13-00529-s001.zip › healthcare-3453075-supplementary.pdf]

Supplementary Material

Supplementary Material S1 Most cited papers

Main Autor (Year of Publication)	Article Title	Times Cited, WoS Core	Journal ISO Abbreviation
Kelder et al. (1994)	Longitudinal Tracking Of Adolescent Smoking, Physical-Activity, And Food Choice Behaviors	951	Am. J. Public Health
Stanton et al. (2020)	Depression, Anxiety And Stress During COVID-19: Associations With Changes In Physical Activity, Sleep, Tobacco And Alcohol Use In Australian Adults	823	Int. J. Environ. Res. Public Health
Lacroix et al. (1993)	Maintaining Mobility In Late-Life .2. Smoking, Alcohol-Consumption, Physical-Activity, And Body-Mass Index	409	Am. J. Epidemiol.
Mozaffarian et al. (2012)	Population Approaches To Improve Diet, Physical Activity, And Smoking Habits A Scientific Statement From The American Heart Association	408	Circulation
Garcia-Aymerich et al. (2007)	Regular Physical Activity Modifies Smoking-Related Lung Function Decline And Reduces Risk Of Chronic Obstructive Pulmonary Disease -: A Population-Based Cohort Study	353	Am. J. Respir. Crit. Care Med.
Mazess et al. (1991)	Bone-Density In Premenopausal Women - Effects Of Age, Dietary-Intake, Physical-Activity, Smoking, And Birth-Control Pills	330	Am. J. Clin. Nutr.
Landinwilhelmsen et al. (1994)	Serum Insulin-Like Growth-Factor-I In A Random-Population Sample Of Men And Women - Relation To Age, Sex, Smoking-Habits, Coffee Consumption And Physical-Activity, Blood-Pressure And Concentrations Of Plasma-Lipids, Fibrinogen, Parathyroid-Hormone And Osteocalcin	309	Clin. Endocrinol.
Noble et al. (2015)	Which Modifiable Health Risk Behaviours Are Related? A Systematic Review Of The Clustering Of Smoking, Nutrition, Alcohol And Physical Activity ('SNAP') Health Risk Factors	289	Prev. Med.

Koh-Banerjee et al. (2003)	Prospective Study Of The Association Of Changes In Dietary Intake, Physical Activity, Alcohol Consumption, And Smoking With 9-Y Gain In Waist Circumference Among 16,587 US Men	265	Am. J. Clin. Nutr.
Lahti-Koski et al. (2002)	Associations Of Body Mass Index And Obesity With Physical Activity, Food Choices, Alcohol Intake, And Smoking In The 1982-1997 FINRISK Studies	247	Am. J. Clin. Nutr.
Adams & Nettle (2009)	Time Perspective, Personality And Smoking, Body Mass, And Physical Activity: An Empirical Study	210	Br. J. Health Psychol.
Ferrucci et al. (1999)	Smoking, Physical Activity, And Active Life Expectancy	208	Am. J. Epidemiol.
Enger et al. (1977)	High-Density Lipoproteins (Hdl) And Physical-Activity - Influence Of Physical Exercise, Age And Smoking On Hdl-Cholesterol And Hdl-1 Total Cholesterol Ratio	197	Scand. J. Clin. Lab. Invest.
Jorgensen (1989)	Gall-Stones In A Danish Population - Relation To Weight, Physical-Activity, Smoking, Coffee Consumption, And Diabetes-Mellitus	195	Gut
Emmons et al. (1994)	Mechanisms In Multiple Risk Factor Interventions - Smoking, Physical-Activity, And Dietary-Fat Intake Among Manufacturing Workers	188	Prev. Med.
Cerhan et al. (1997)	Association Of Smoking, Body Mass, And Physical Activity With Risk Of Prostate Cancer In The Iowa 65+ Rural Health Study (United States)	186	Cancer Causes Control
Kaczynski et al. (2008)	Smoking And Physical Activity: A Systematic Review	177	Am. J. Health Behav.
Paavola et al. (2004)	Smoking, Alcohol Use, And Physical Activity: A 13-Year Longitudinal Study Ranging From Adolescence Into Adulthood	167	J. Adolesc. Health
Slattery et al. (1992)	Associations Of Body-Fat And Its Distribution With Dietary-Intake, Physical-Activity, Alcohol, And Smoking In Blacks And Whites	160	Am. J. Clin. Nutr.
Ng et al. (2020)	Smoking, Drinking, Diet And Physical Activity - Modifiable Lifestyle Risk Factors And Their Associations With Age To First Chronic Disease	153	Int. J. Epidemiol.
Milne-Ives et al. (2020)	Mobile Apps For Health Behavior Change In Physical Activity, Diet, Drug And Alcohol Use, And Mental Health: Systematic Review	149	JMIR mHealth uHealth

Seidell et al. (1991)	Body-Fat Distribution In Relation To Physical-Activity And Smoking-Habits In 38-Year-Old European Men - The European Fat Distribution Study	146	Am. J. Epidemiol.
American Diabetes Association (2014)	Foundations Of Care: Education, Nutrition, Physical Activity, Smoking Cessation, Psychosocial Care, And Immunization	137	Diabetes Care
Shiels et al. (2009)	Association Of Cigarette Smoking, Alcohol Consumption, And Physical Activity With Sex Steroid Hormone Levels In US Men	132	Cancer Causes Control
Steptoe et al. (2001)	The Impact Of Behavioral Counseling On Stage Of Change In Fat Intake, Physical Activity, And Cigarette Smoking In Adults At Increased Risk Of Coronary Heart Disease	132	Am. J. Public Health
Afshin et al. (2016)	Information Technology And Lifestyle: A Systematic Evaluation Of Internet And Mobile Interventions For Improving Diet, Physical Activity, Obesity, Tobacco, And Alcohol Use	131	J. Am. Heart Assoc.
Weischer et al. (2014)	Telomere Shortening Unrelated To Smoking, Body Weight, Physical Activity, And Alcohol Intake: 4,576 General Population Individuals With Repeat Measurements 10 Years Apart	129	PLoS Genet.
Rosario et al. (2014)	Sexual Orientation Disparities In Cancer-Related Risk Behaviors Of Tobacco, Alcohol, Sexual Behaviors, And Diet And Physical Activity: Pooled Youth Risk Behavior Surveys	122	Am. J. Public Health
Oenema et al. (2008)	Efficacy And Use Of An Internet-Delivered Computer-Tailored Lifestyle Intervention, Targeting Saturated Fat Intake, Physical Activity And Smoking Cessation: A Randomized Controlled Trial	119	Ann. Behav. Med.
Troisi et al. (1991)	Cigarette-Smoking, Dietary-Intake, And Physical-Activity - Effects On Body-Fat Distribution - The Normative Aging Study	117	Am. J. Clin. Nutr.
Vancampfort et al. (2013)	Relationships Between Physical Fitness, Physical Activity, Smoking And Metabolic And Mental Health Parameters In People With Schizophrenia	112	Psychiatry Res.
Moreno-Gomez et al. (2012)	Clustering Of Lifestyle Factors In Spanish University Students: The Relationship Between Smoking,	106	Public Health Nutr.

	Alcohol Consumption, Physical Activity And Diet Quality		
Larson et al. (2014)	Adolescent Consumption Of Sports And Energy Drinks: Linkages To Higher Physical Activity, Unhealthy Beverage Patterns, Cigarette Smoking, And Screen Media Use	105	J. Nutr. Educ. Behav.
Stolzenberg-Solomon et al. (2022)	A Prospective Study Of Medical Conditions, Anthropometry, Physical Activity, And Pancreatic Cancer In Male Smokers (Finland)	105	Cancer Causes Control
Rask-Andersen et al. (2017)	Gene-Environment Interaction Study For BMI Reveals Interactions Between Genetic Factors And Physical Activity, Alcohol Consumption And Socioeconomic Status	103	PLoS Genet.
Emmons et al. (1999)	The Working Healthy Project: A Worksite Health-Promotion Trial Targeting Physical Activity, Diet, And Smoking	103	J. Occup. Environ. Med.
Nierkens et al. (2013)	Effectiveness Of Cultural Adaptations Of Interventions Aimed At Smoking Cessation, Diet, And/Or Physical Activity In Ethnic Minorities. A Systematic Review	99	PLoS One
Bull et al. (2014)	Are Interventions For Low-Income Groups Effective In Changing Healthy Eating, Physical Activity And Smoking Behaviours? A Systematic Review And Meta-Analysis	97	BMJ Open
Santos et al. (2007)	Alcohol Intake, Smoking, Sleeping Hours, Physical Activity And The Metabolic Syndrome	96	Prev. Med.
Luo et al. (2007)	Body Mass Index, Physical Activity And The Risk Of Pancreatic Cancer In Relation To Smoking Status And History Of Diabetes: A Large-Scale Population-Based Cohort Study In Japan - The JPHC Study	94	Cancer Causes Control
Colbert et al. (2011)	Physical Activity In Relation To Cancer Of The Colon And Rectum In A Cohort Of Male Smokers	93	Cancer Epidemiol. Biomarkers Prev.
Rohrmann et al. (2005)	Association Of Cigarette Smoking, Alcohol Consumption And Physical Activity With Lower Urinary Tract Symptoms In Older American Men: Findings From The Third National Health And Nutrition Examination Survey	92	BJU Int.

Lowry et al. (2002)	Weight Management Goals And Practices Among US High School Students: Associations With Physical Activity, Diet, And Smoking	91	J. Adolesc. Health
Schnohr et al. (2004)	Does Educational Level Influence The Effects Of Smoking, Alcohol, Physical Activity, And Obesity On Mortality? : A Prospective Population Study	88	Scand. J. Public Health
Audrain-Mcgovern et al. (2003)	Smoking Progression And Physical Activity	87	Cancer Epidemiol. Biomarkers Prev.
Godsland et al. (1998)	Associations Of Smoking, Alcohol And Physical Activity With Risk Factors For Coronary Heart Disease And Diabetes In The First Follow-Up Cohort Of The Heart Disease And Diabetes Risk Indicators In A Screened Cohort Study (HDDRISC-1)	87	J. Intern. Med.
Kawachi et al. (1996)	Can Physical Activity Minimize Weight Gain In Women After Smoking Cessation?	87	Am. J. Public Health
Cohen et al. (2003)	Smoking, Physical Activity And Breakfast Consumption Among Secondary School Students In A Southwestern Ontario Community	86	Can. J. Public Health-Rev. Can. Sante Publ.
Shi et al. (2013)	Physical Activity, Smoking, And Alcohol Consumption In Association With Incidence Of Type 2 Diabetes Among Middle-Aged And Elderly Chinese Men	85	PLoS One
Oosterveen et al. (2017)	A Systematic Review Of Ehealth Behavioral Interventions Targeting Smoking, Nutrition, Alcohol, Physical Activity And/Or Obesity For Young Adults	84	Prev. Med.
Klesges et al. (1990)	Smoking Status - Effects On The Dietary-Intake, Physical-Activity, And Body-Fat Of Adult Men	84	Am. J. Clin. Nutr.
Latifovic et al. (2016)	The Influence Of Alcohol Consumption, Cigarette Smoking, And Physical Activity On Leukocyte Telomere Length	78	Cancer Epidemiol. Biomarkers Prev.
Vankim et al. (2010)	Understanding Young Adult Physical Activity, Alcohol And Tobacco Use In Community Colleges And 4-Year Post-Secondary Institutions: A Cross-	78	BMC Public Health

	Sectional Analysis Of Epidemiological Surveillance Data		
Haenle et al. (2006)	Overweight, Physical Activity, Tobacco And Alcohol Consumption In A Cross-Sectional Random Sample Of German Adults	78	BMC Public Health
Donahue et al. (1999)	Cigarette Smoking, Alcohol Use, And Physical Activity In Relation To Serum Leptin Levels In A Multiethnic Population: The Miami Community Health Study	76	Ann. Epidemiol.
Prochaska et al. (2008)	Physical Activity As A Strategy For Maintaining Tobacco Abstinence: A Randomized Trial	74	Prev. Med.
Vergnaud et al. (2012)	Fruit And Vegetable Consumption And Prospective Weight Change In Participants Of The European Prospective Investigation Into Cancer And Nutrition-Physical Activity, Nutrition, Alcohol, Cessation Of Smoking, Eating Out Of Home, And Obesity Study	71	Am. J. Clin. Nutr.
Anton & Miller (2005)	Do Negative Emotions Predict Alcohol Consumption, Saturated Fat Intake, And Physical Activity In Older Adults?	71	Behav. Modificat.
Yach et al. (2005)	Improving Diet And Physical Activity: 12 Lessons From Controlling Tobacco Smoking	71	BMJ-British Medical Journal
Jayasekara et al. (2018)	Associations Of Alcohol Intake, Smoking, Physical Activity And Obesity With Survival Following Colorectal Cancer Diagnosis By Stage, Anatomic Site And Tumor Molecular Subtype	68	Int. J. Cancer
Cosnes (2010)	Smoking, Physical Activity, Nutrition And Lifestyle: Environmental Factors And Their Impact On IBD	67	Dig. Dis.
Haveman-Nies (2003)	Relation Of Dietary Quality, Physical Activity, And Smoking Habits To 10-Year Changes In Health Status In Older Europeans In The SENECA Study	66	Am. J. Public Health
